# Temperature Dependent near Infrared Spectroscopy of Electron Irradiated Ceria Single Crystals

**DOI:** 10.3390/ma17163892

**Published:** 2024-08-06

**Authors:** Jean-Marc Costantini, Keevin Béneut, Maxime Guillaumet, Gérald Lelong

**Affiliations:** 1 Université Paris-Saclay, CEA, Service de Recherche en Matériaux et Procédés Avancés, 91191 Gif-sur-Yvette, France; 2 IMPMC, de Physique des Matériaux et de Cosmochimie, Institut de Minéralogie, IRD, UMR CNRS 7590, Muséum National d’Histoire Naturelle, Sorbonne Université, 75005 Paris, France; keevin.beneut@sorbonne-universite.fr (K.B.); maxime.guillaumet@sorbonne-universite.fr (M.G.); gerald.lelong@sorbonne-universite.fr (G.L.)

**Keywords:** cerium dioxide, electron irradiation, near infrared spectroscopy

## Abstract

The FTIR absorption bands of virgin and electron-irradiated CeO_2_ single crystals were measured from 20 K to 500 K between 4000 cm^−1^ and 12,000 cm^−1^ (~830 nm to 2500 nm). Three broad bands centered at about 6100 cm^−1^ (~0.75 eV), 7000 cm^−1^ (~0.87 eV), and 10,500 cm^−1^ (~1.3 eV) were recorded above 100 K for the 2.5 MeV electron energy. Two smaller bands at about 4300 cm^−1^ (~0.53 eV) and 5500 cm^−1^ (~0.68 eV) were also recorded below 100 K. Similar broad bands centered at about 4100 cm^−1^ (~0.52 eV), 6400 cm^−1^ (~0.79 eV), 7600 cm^−1^ (~0.94 eV), and 10,500 cm^−1^ (~1.3 eV) are also found for the 1.4 MeV electron energy above 300 K. The evolution of these absorption bands was followed as a function of temperature. The plots of band intensity ratios show a thermally activated process corresponding to the ionization of the deep electronic levels of point defects in the band gap of ceria of ~26,000 cm^−1^ (~3.2 eV). These five bands are assigned to the different charge states (0, −1, −2, −3, −4) of the Ce vacancies produced by elastic collisions above 1.0 MeV.

## 1. Introduction

There has been quite a significant development of solid oxide fuel cell (SOFC) materials over the last ten years [1] and particularly on ceria (CeO_2_), either undoped or doped with trivalent rare-earth elements [2]. Oxygen vacancy formation by charge compensation is actually envisioned so as to increase the conductivity of ceria [2]. In this respect, the knowledge of defect formation by other means like charged particle irradiation can be instrumental for this purpose. Actually, electron irradiation in the 1 MeV energy range is a relevant method for inducing a homogeneous distribution of point defects by elastic collision processes in a large volume owing to the large range of these fast electrons.

Various experimental techniques were applied to study the defect formation by irradiation of ceria such as UV–visible spectroscopy [3], near infra-red (NIR) spectroscopy [4], cathodo-luminescence spectroscopy [5], electron paramagnetic spectroscopy (EPR) [6], X-ray photoelectron spectroscopy (XPS) [7], X-ray absorption spectroscopy (XANES) [8], and positron annihilation spectroscopy (PAS) [9]. One key result is the reduction of Ce^4+^ ions by either elastic or inelastic processes after electron [6] or ion irradiations [3].

Room-temperature NIR spectra of electron-irradiated ceria single crystals have also suggested the formation of cerium vacancies for electron energies above 1.0 MeV [4]. The scope of the present paper is to extend these NIR spectroscopy data as a function of temperature from 20 K to 500 K in view to support these conclusions. We provide new results on ceria single crystals, which are not commercially available. To the best of our knowledge, no FTIR spectra of irradiated ceria were recorded as a function of temperature whereas most data were obtained at room temperature. Amongst other experimental techniques, optical absorption spectroscopy allows a most accurate characterization of point defects in solids. The temperature dependence of the NIR band intensities is consistent with the thermal ionization of the deep defect levels associated to cerium vacancies in the band gap of ceria for 1.4 MeV and 2.5 MeV electron irradiations. This work brings forth a better knowledge of point defect characterization in ceria. 

## 2. Experimental Procedure

We have used 1 mm-long CeO_2_ single crystals with a cubic fluorite structure (see the pictures in [10]), which were grown at the Oak Ridge National Laboratory (L. Boatner). Electron irradiations of the single crystals for the 1.4 MeV and 2.5 MeV energy were carried out near room temperature up to fluences of 4.2 × 10^16^ cm^−2^ and 1.5 × 10^16^ cm^−2^, respectively. The crystals turned from a light blue color to a deep green color. Table 1 displays the irradiation parameters such as the inelastic energy loss and CSDA (continuous slowing down approximation) range of electrons computed with the ESTAR code [11]. The electrons were shot through the target samples and were inducing homogeneous damage in the single crystal thickness.

The NIR measurements were carried out in the transmission mode at normal incidence on a triangular {1 1 1} facet of the single crystals by using a Bruker IFS 66v/S spectrometer (Bruker, Billerica, MA, USA) working under vacuum with a tungsten source, quartz beam splitter, and nitrogen-cooled MCT detector. The single crystals were embedded in a KBr pellet matrix, which is transparent in this IR photon range. The light source was transmitted through a 1 mm pinhole directly in contact with the sample to be sure of the illuminated area. The spectral resolution was of 4 cm^−1^ for scans in the 4000–12,000 cm^−1^ range. The spectra were recorded after cooling from 300 K to 20 K then after warming up to 500 K step by step (see the temperatures in Table 2 and Table 3) thanks to a liquid–helium flow cryostat kept under a high vacuum of 10^−6^ mbar.

## 3. Results

**Figure 1:**
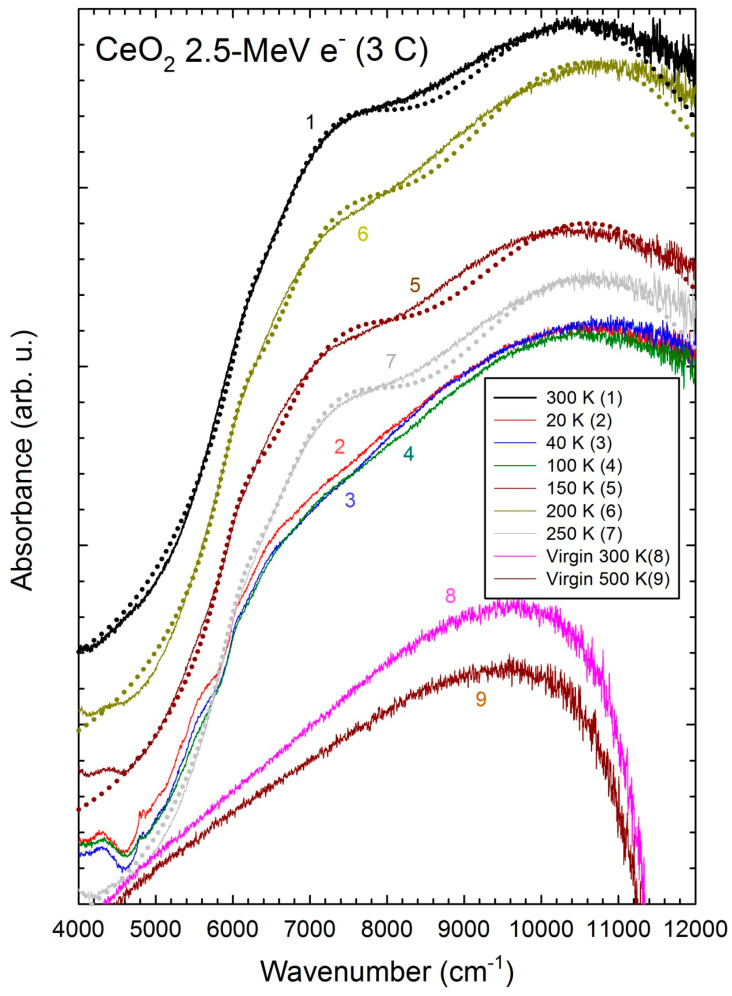
NIR absorption spectra of the 2.5 MeV electron-irradiated ceria single crystal for various temperatures. The virgin sample spectra for 300 K and 500 K are also shown. The dotted lines are fitted curves with Gaussian profiles.

**Figure 2:**
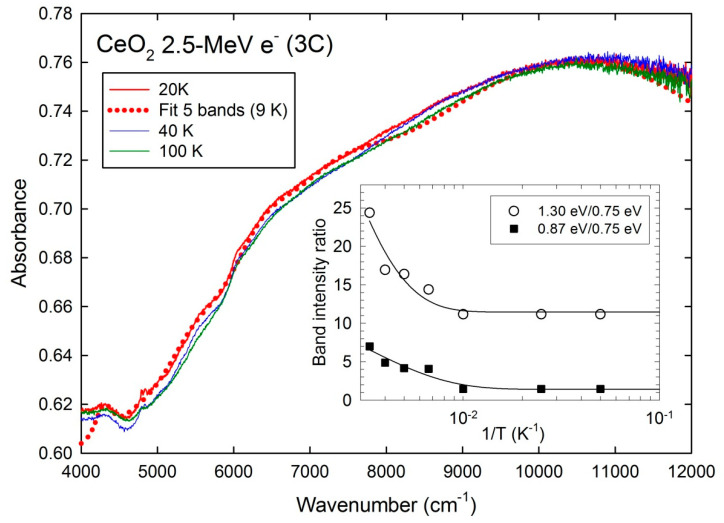
NIR absorption spectra of the 2.5 MeV electron-irradiated ceria single crystal for various temperatures. The dotted lines are fitted curves with Gaussian profiles. Inset: maximum absorbance ratios of the 1.3 eV and 0.75 eV bands and 0.87 eV and 0.75 eV bands as a function of the reciprocal temperature. The solid lines are least-square fits of data with Equation (1).

The NIR absorption spectra of the single crystal irradiated with 2.5 MeV electrons are fitted with Gaussian profiles for the various temperatures from 20 K to 300 K (Figure 1 and Figure 2). Three broad bands centered at about 6100 cm^−1^ (~0.75 eV), 7000 cm^−1^ (~0.87 eV), and 10,500 cm^−1^ (~1.3 eV) are used for the temperatures above 100 K, whereas two extra smaller bands at about 4300 cm^−1^ (~0.53 eV) and 5500 cm^−1^ (~0.68 eV) are added for the spectra at lower temperatures (Table 2). The widths as given from the standard deviations (σ) of the Gaussian curves are almost the same regardless of temperature. The smaller bands centered at about 4300 cm^−1^ and 5500 cm^−1^ do not show significant variation versus temperature, whereas the two other bands at about 7000 cm^−1^ and 10,500 cm^−1^ increase in intensity with temperature. No such bands are found in the FTIR absorption spectra of the virgin single crystal recorded at 300 K and 500 K (Figure 1). Subtraction of these reference spectra with different shapes from those of irradiated samples is not relevant. Therefore, we have considered the evolution of the fitted band intensity values as a function of temperature. 

The maximum absorbance of the two latter bands at about 7000 cm^−1^ and 10,500 cm^−1^ is plotted with reference to the 6100 cm^−1^ band (~0.75 eV) as a function of reciprocal temperature ($\frac{1}{T}$) (Figure 2, inset). The ratios of band intensities (R) are least-square fitted with the following equation:

(1) $$R=R_{0}+K\;exp\;(-\frac{E^{*}}{k_{B}T})$$

where R_0_ and (R_0_ + K) are the asymptotic values for low and high temperatures, respectively, E* is an effective activation energy, and k_B_ the Boltzmann’s constant. Fitted R_0_-values of about 1.4 and 11.4, and k-values of about 15 and 95 are obtained for the intensity ratios of the 0.87 eV and 0.75 eV bands and of the 1.3 eV and 0.75 eV bands, respectively. Low fitted values of about 0.03 and 0.06 eV for E* are found for these two ratios, respectively. However, the real activation energy is E = log K − E*, which gives E = 1.14 eV and E = 1.92 eV, for the (0.87 eV/0.75 eV) and (1.3 eV/0.75 eV) ratios, respectively. No significant differences are found by considering the integrated intensity ratios since the band widths do not vary significantly with temperature (Table 2).

**Figure 3:**
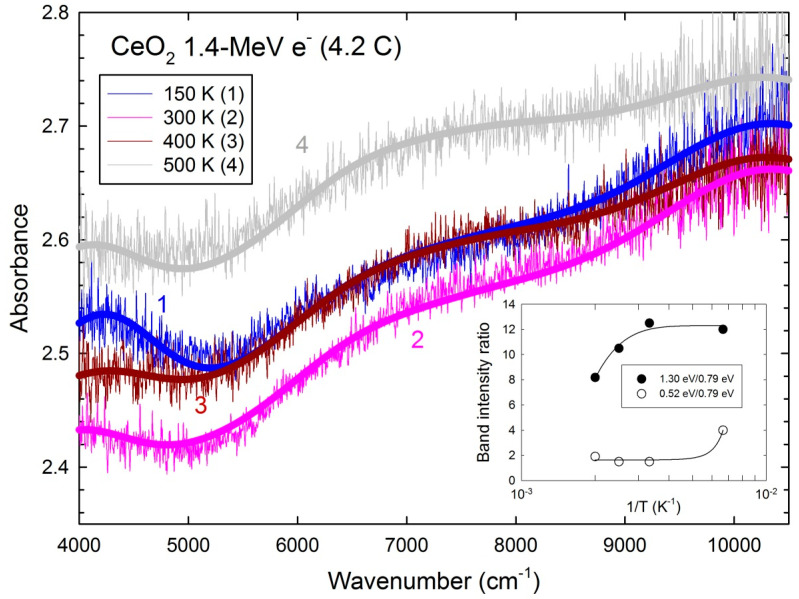
NIR absorption spectra of the 1.4 MeV electron-irradiated ceria single crystal for various temperatures. The dotted lines are fitted curves with Gaussian profiles. Inset: maximum absorbance ratios of the 1.30 eV and 0.79 eV bands and 0.52 eV and 0.79 eV bands as a function of the reciprocal temperature. The solid lines are least-square fits with an exponential rise to saturation for the (1.30 eV/0.79-eV) ratio and with an exponential rise for the (0.52 eV/0.79 eV) ratio.

For the 1.4-MeV electron energy, similar four broad bands centered 4100 cm^−1^ (~0.52 eV), 6400 cm^−1^ (~0.79 eV), 7600 cm^−1^ (~0.94 eV), and 10,500 cm^−1^ (~1.3 eV) are deduced from the fits of the spectra from 300 K to 500 K (Figure 3). The higher background level likely arises from the enhanced light scattering by the smaller single crystal. The intensity ratios of the 0.52 eV and 0.79 eV bands and the 1.3 eV and 0.79 eV bands, respectively, are plotted versus $\frac{1}{T}$ (Figure 3, inset). This shows a decrease of both the 0.52 eV and 1.3 eV bands vs. temperature, whereas the intensity of the 0.79 eV band shows no change as a function of temperature (Table 3). 

## 4. Discussion

**Figure 4:**
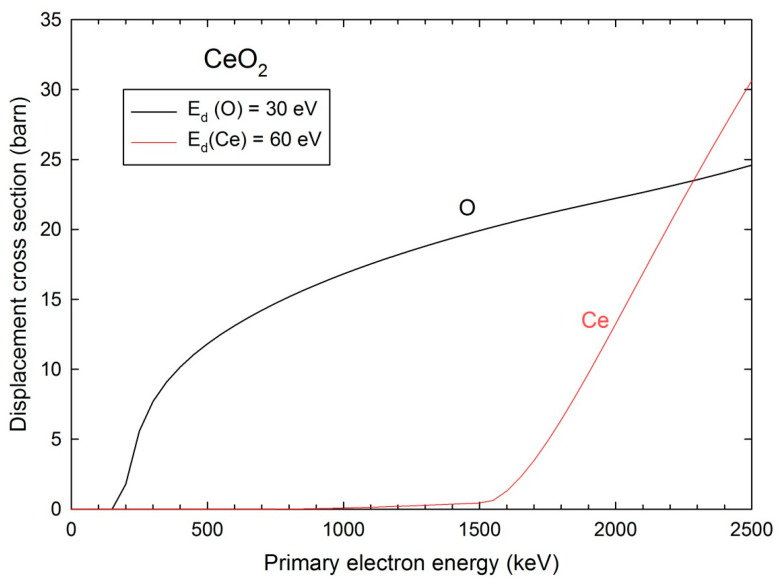
Displacement cross sections of Ce and O atoms versus electron energy.

The present results for 1.4 MeV and 2.5 MeV electron irradiation for temperatures from 20 K to 500 K are rather consistent with the previous data for the 2.5 MeV electron-irradiated sample recorded at room temperature showing five broad absorption bands in the NIR range centered at about 3600 cm^−1^, 4100 cm^−1^, 4600 cm^−1^, 6000 cm^−1^, and 7100 cm^−1^ (i.e., ~0.44, 0.51, 0.57, 0.74, and 0.88 eV) [4]. The band centered at about 10,500 cm^−1^ (~1.3 eV) found in the present data for both electron energies could not be resolved in the previous spectra due to the large background noise above 9500 cm^−1^. These five bands were previously assigned to the optical transitions of the cerium vacancies (V_Ce_) with five possible charge states (0, −1, −2, −3, −4). Actually, Ce atom displacement by elastic collisions takes place for electron energies above 1.0 MeV [14], as seen from the displacement cross section (σ_d_) values computed with the SMOTT/POLY code [12] for a threshold displacement energy of E_d_ (Ce) = 60 eV [13] (Figure 4). This is consistent with the color change from the light blue to deep green color for the 1.4 MeV and 2.5 MeV electron irradiations [10], whereas no change in sample color is seen for the 1.0 MeV energy. The σ_d_ value for Ce atoms is quite lower for the 1.4 MeV electron energy than for 2.5 MeV, but it is partly compensated by the higher fluence (ϕ) that can induce a sufficient number of Ce vacancies (Table 1). Instead, no strong variation of σ_d_ for O atoms is computed in the same electron energy range for E_d_ (Ce) = 30 eV [15] (Table 1) (Figure 4). The total number of displacements per Ce atom is dpa (Ce) = σ_d_ ϕ ~ 1.5 × 10^−8^ for the 1.4 MeV electrons, whereas dpa (Ce) ~ 9 × 10^−7^ for 2.5 MeV. This gives concentrations of displaced Ce atoms of 0.015 ppm and 0.9 ppm, respectively. However, no clear differences in the absorption band intensities are found at 300 K between these two electron energies. It is to be noted that band intensities not only depend on intrinsic factors of the optical absorbance such as the number of defects, defect level populations, and oscillator strengths of dipolar transitions, but also on the light scattering depending on the sample shape. It is actually seen that the background level is much larger for the 1.4 MeV energy spectra (Figure 3) than for the 2.5 MeV ones (Figure 1 and Figure 2). Therefore, the assessment of band intensities is more challenging for the former case. 

The activation energies E of about 1.1 eV and 1.9 eV deduced from the R values for the 2.5 MeV electron energy (Figure 2, inset) are rather consistent with a thermally activated ionization process of these defect levels located in the band gap of CeO_2_. The deviations with the IR absorption bands at 0.87 eV and 1.3 eV are likely due to the uncertainties on the fitted band intensities. Those transitions are well below the 2p–4f optical gap of ~3.2 eV (~25,800 cm^−1^) and deep in the 2p–5d gap of ~5.5 eV (~44,000 cm^−1^) [16]. The asymptotic R_0_ values of about 1 and 10 for T ≤ 100 K correspond to the equilibrium population of the electronic levels at low temperature which is lower for the 0.87 eV band than for the deeper 1.3 eV band. This is also consistent with a thermally activated ionization process. Accordingly, the intensities of the shallower bands exhibit fewer variations versus temperature. There is an opposite temperature dependence of R values for the 1.4 MeV energy corresponding to the thermal excitation of the deeper 1.3 eV level above 300 K. There is an inversion of the equilibrium populations at high temperatures.

We assume that the absorption band intensities are directly proportional to the populations of the defect levels. The number of electrons with up and down spins in the i-th levels can be written as:

(2) $$n_{i}=2\;N_{i}\;f_{i}=2\;{N_{i}\;[1+exp\;(\frac{E_{i}-E_{F}}{k_{B}T})]}^{-1}$$

where $N_{i}$ is the number of defect levels, $f_{i}\;$ is the occupation number given by the Fermi–Dirac statistics, $E_{i}$ is the energy of the i-th level (as measured above the valence band edge), and $E_{F}$ is the Fermi level energy. For intrinsic or lightly doped CeO_2_, $E_{F}$ should be near 3 eV (i.e., about half the 2p–5d band gap, E_G_) close to the 4f level position. The electron number ratio ($\rho_{i,j}$) between two levels in numbers of N_i_ and N_j_ is given by:

(3) $$\rho_{i,j}=\frac{N_{i}f_{i}}{N_{j}f_{j}}=\frac{{N_{i}\;[1+exp\;(\frac{E_{i}-E_{F}}{k_{B}T})]}^{-1}}{{N_{j}\;[1+exp\;(\frac{E_{j}-E_{F}}{k_{B}T})]}^{-1}}$$

We surmise that these levels are occupied V_Ce_ hole centers for $E_{i}<E_{F}$ and $E_{j}<E_{F}$ [13]. For high temperatures, when $\frac{1}{T}\to 0$, $\rho_{i,j}\to \frac{N_{i}}{N_{j}}\;$ and the band intensity ratio is of $R_{i,j}\propto \rho_{i,j}\propto \frac{N_{i}}{N_{j}}$. As a result, the high-temperature asymptotic values (R_0_ + k), that are not reached in our measurements correspond to the ratio of defect numbers. For low temperatures, when $\frac{1}{T}\to +\infty$, $R_{i,j}\propto \;\rho_{i,j}\to \frac{N_{i}\;exp\;(\frac{E_{j}-E_{F}}{k_{B}T})}{N_{j}\;exp\;(\frac{E_{i}-E_{F}}{k_{B}T})}$. The low-temperature asymptotic values of $R_{0}$ give the ratio of the equilibrium populations of the defect levels as given by the Boltzmann factors that are assigned to the various charge states of $V_{C_{e}}$. Moreover, the numbers of these Ce vacancy levels are bounded by the total number of residual defects given by the total number of displaced Ce atoms (N_d_ (Ce)), such as: $\sum_{1}^{5}N_{i}\leq N_{d}\;(C_{e})$, by taking into account possible Ce Frenkel pair recombination. This number can be estimated from the dpa value as:

(4) $$N_{d}\left(C_{e}\right)=dpa\left(C_{e}\right)(4\;\frac{V}{a_{0}^{3}})$$

where a_0_ = 0.5411 nm is the lattice parameter of CeO_2_ with four Ce atoms per unit cell of the cubic fluorite structure, and V ~ 1 mm^3^ is the single crystal volume. This gives N_d_ (Ce) ~ 3.8 × 10^11^ for the 1.4 MeV electron irradiation, and N_d_ (Ce) ~ 2.3 × 10^13^ for the 2.5 MeV electron irradiation.

According to the above analysis, the absorption band intensities are directly proportional to the defect level populations and number of defects. For 20 K ≤ T ≤ 300 K, the shallower levels corresponding to the 0.75 eV band are more depleted by ionization than the deeper levels corresponding to the 0.87 eV and 1.3 eV bands. This induces an increase of the (0.87 eV/0.75 eV) and (1.3 eV/0.75 eV) ratios when temperature increases (Figure 2, inset). By contrast, for 300 K ≤ T ≤ 500 K, the deeper levels start to be ionized and the intensity ratio of the 1.3 eV and 0.79 eV bands decreases with temperature (Figure 3, inset). The ratio of 0.52 eV and 0.79 eV bands decays to an asymptotic behavior for T > 300 K. 

**Figure 5:**
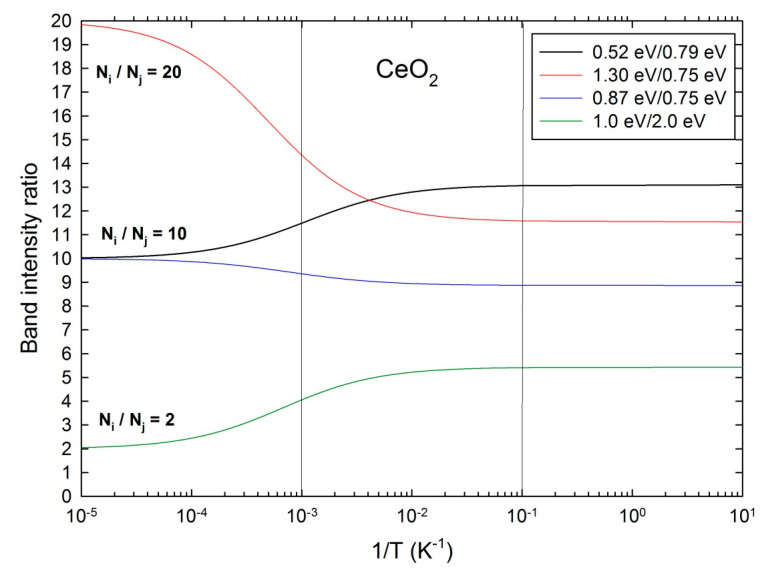
Calculated intensity ratios of the 0.52 eV and 0.79 eV bands and 0.87 eV and 0.75 eV bands for $\frac{N_{i}}{N_{j}}=10$ and of the 1.3 eV and 0.75 eV bands for $\frac{N_{i}}{N_{j}}=20$ as a function of the reciprocal temperature for a Fermi energy of E_F_ = 3.0 eV. The curve for 1.0 eV and 2.0 eV energy levels is also computed for $\frac{N_{i}}{N_{j}}=2$ and E_F_ = 3.0 eV. The vertical lines mark the range of temperature of measurements.

Plots of the (0.87 eV/0.75 eV) and (1.3 eV/0.75 eV) intensity ratios are calculated vs. $\frac{1}{T}$ from Equation (3) with E_F_ = 3 eV, and for $\frac{N_{j}}{N_{i}}=10$ and $\frac{N_{j}}{N_{i}}=20$, respectively (Figure 5). The temperature range of measurements are marked by two vertical limits showing a similar behavior as the experimental plots in this range (Figure 2, inset), with the two asymptotic behaviors at high temperatures for T > 1000 K and low temperatures for T < 100 K, even though the values of ratios are different. For the sake of comparison, the curve for energy levels of 1.0 eV and 2.0 eV is also computed for E_F_ = 3 eV and $\frac{N_{i}}{N_{j}}=2$ (Figure 5). For $E_{i}<E_{j}$, $R_{i,j}$ is a decreasing function of T, whereas for $E_{i}>E_{j}$, $R_{i,j}$ is an increasing function. The plot of the (0.52 eV/0.79 eV) intensity ratio for $\frac{N_{j}}{N_{i}}=10$ shows an opposite variation as the experimental data (Figure 3, inset). A fit of data with Equation (3) would need to adjust five free parameters that are not fully independent, since the chemical potential or Fermi energy (E_F_) depends on the numbers (N_i_ and N_j_) and energies (E_i_ and E_j_) of the electronic levels [13]. We chose E_F_ = 3 eV, since $E_{F}=\frac{1}{2}E_{G}$ for an intrinsic material, but the doping by point defects definitely shifts the Fermi level off the mid-gap position. For extrinsic semiconductors, the Fermi level is the electron chemical potential depending on the donor and acceptor levels in the band gap [13].

Since Ce vacancies are hole centers, E_F_ is likely pushed down in the band gap [13]. Due to the large Hubbard U(O_2p_) value, ranging from 4 to 5.5 eV in CeO_2_, the holes of V_Ce_’’’’ are localized on the neighboring oxygen atoms [17]. This hole-center state is mainly composed of empty O_2p_ orbitals. We assume that these weak intensity bands in the NIR range could be assigned to 2p (O) → 2p (V_Ce_) parity-forbidden electric dipole transitions according to Laporte’s selection rules. The present results confirm that the NIR absorption bands of electron-irradiated ceria can be assigned to Ce vacancies induced by elastic collisions above the 1.0 MeV energy.

## 5. Conclusions

The FTIR spectra of a CeO_2_ single crystal were measured between 4000 and 12,000 cm^−1^ from 20 K to 500 K after 1.4 MeV and 2.5 MeV electron irradiations near 300 K. Five broad bands centered at about 4300 cm^−1^ (~0.53 eV), 5500 cm^−1^ (~0.68 eV), 6100 cm^−1^ (~0.75 eV), 7000 cm^−1^ (~0.87 eV), and 10,500 cm^−1^ (~1.3 eV) are recorded. The evolution of these absorption bands as a function of temperature is analyzed by a thermally activated ionization process of the electronic levels assigned to the various charge states (0, −1, −2, −3, −4) of the Ce vacancies produced by elastic collisions.

## Figures and Tables

**Table 1:** Parameters of electron irradiations of CeO_2_ with mass density of 7.215 g cm^−3^: incident energy (E), fluence (ϕ), electronic energy loss (S_e_), and CSDA range computed with the ESTAR code (for the ionization energy I = 407.6 eV) [11], and displacement cross sections (σ_d_) for Ce and O atoms in barn unit (b) computed with the SMOTT/POLY code [12] for the threshold displacement energies of E_d_ = 60 eV and E_d_ = 30 eV [13], respectively (Figure 4).

E (MeV)	ϕ (cm^−2^)	S_e_ (MeV µm^−1^)	σ_d_ (Ce) (b)	σ_d_ (O) (b)	Range (mm)
1.0		9.0 × 10^−4^	0.079	16.81	8.4
1.4	4.2 × 10^16^	9.2 × 10^−4^	0.36	19.37	1.5
2.5	3.0 × 10^16^	9.9 × 10^−4^	29.91	20.40	2.5

**Table 2:** Fitted parameters of the NIR absorption bands for the single crystal irradiated with 2.5 MeV electrons for the fluence of 3.0 × 10^16^ cm^−2^ (irradiation dose of 3 C): band center, standard deviation (σ), and absorbance at maximum of the Gaussian profiles used for the fits.

T (K)	20	40	100	150	200	250	300
Band center 1 (cm^−1^)	4300	4300	4300				
σ (cm^−1^)	100	100	100				
Absorbance 1	0.01	0.01	0.01				
Band center 2 (cm^−1^)	5500	5500	5500				
σ (cm^−1^)	300	300	300				
Absorbance 2	0.012	0.012	0.012				
Band center 3 (cm^−1^)	6300	6300	6300	6100	6100	6100	6300
σ (cm^−1^)	350	350	350	250	250	250	350
Absorbance 3	0.017	0.017	0.017	0.0125	0.0125	0.0115	0.017
Band center 4 (cm^−1^)	7100	7100	7100	7000	7000	7000	7000
σ (cm^−1^)	700	700	700	950	900	900	900
Absorbance 4	0.025	0.025	0.025	0.051	0.052	0.056	0.056
Band center 5 (cm^−1^)	10,500	10,500	10,500	10,600	10,600	10,600	10,400
σ (cm^−1^)	3500	3500	3500	3000	3000	3000	3000
Absorbance 5	0.19	0.19	0.19	0.18	0.205	0.195	0.195

**Table 3:** Fitted parameters of the NIR absorption bands for the single crystal irradiated with 1.4 MeV electrons for the fluence of 4.2 × 10^16^ cm^−2^ (irradiation dose of 4.2 C): band center, standard deviation (σ), and absorbance at maximum of the Gaussian profiles used for the fits.

T (K)	150	300	400	500
Band center 1 (cm^−1^)	4200	4000	4200	4100
σ (cm^−1^)	450	450	500	450
Absorbance 1	0.08	0.03	0.03	0.04
Band center 2 (cm^−1^)	6400	6400	6400	6400
σ (cm^−1^)	500	600	600	600
Absorbance 2	0.02	0.02	0.02	0.02
Band center 3 (cm^−1^)	7600	7600	7600	7600
σ (cm^−1^)	1300	1300	1300	1300
Absorbance 3	0.13	0.13	0.13	0.13
Band center 4 (cm^−1^)	10,500	10,500	10,500	10,500
σ (cm^−1^)	1300	1300	1300	1300
Absorbance 4	0.24	0.25	0.21	0.18

## Data Availability

The original contributions presented in the study are included in the article, further inquiries can be directed to the corresponding author.
